# Stable Frequencies of HLA-C^*^03:04/Peptide-Binding KIR2DL2/3^+^ Natural Killer Cells Following Vaccination

**DOI:** 10.3389/fimmu.2018.02361

**Published:** 2018-10-17

**Authors:** Maja Christiane Ziegler, Ferran Borràs Grañana, Wilfredo F. Garcia-Beltran, Julian Schulze zur Wiesch, Christian Hoffmann, Anne Rechtien, Sebastian Lunemann, Marcus Altfeld

**Affiliations:** ^1^Department of Virus Immunology, Heinrich Pette Institute, Leibniz Institute for Experimental Virology, Hamburg, Germany; ^2^Ragon Institute of MGH, MIT and Harvard, Cambridge, MA, United States; ^3^First Department of Medicine, University Medical Center Hamburg-Eppendorf, Hamburg, Germany; ^4^ICH Study Center, Infektionsmedizinisches Centrum Hamburg, Hamburg, Germany; ^5^Partner Site Hamburg-Lübeck-Borstel-Riems, German Center for Infection Research, Hamburg, Germany

**Keywords:** HLA, KIR, NK cells, HCV, HIV, YFV vaccine

## Abstract

Inhibitory KIRs play a central role in regulating NK cell activity. KIR2DL2/3 bind to HLA-C molecules, but the modulation of these interactions by viral infections and presentation of viral epitopes is not well-understood. We investigated whether the frequencies of KIR2DL2/3^+^ NK cells recognizing HLA-C^*^03:04/viral peptide complexes were impacted by YFV vaccination or HIV-1 and HCV infection. *Ex vivo* HLA class I tetramer staining of primary human NK cells derived from YFV-vaccinated individuals, or HIV-1- or HCV-infected individuals revealed that the YFV/HLA-C^*^03:04-NS2A_4−13_-tetramer bound to a larger proportion of KIR2DL2/3^+^ NK cells compared to HIV-1/HLA-C^*^03:04-Gag_296−304_- or HCV/HLA-C^*^03:04-Core_136−144_-tetramers. The YFV/HLA-C^*^03:04-NS2A_4−13_-tetramer also exhibited a stronger avidity to KIR2DL2/3 compared to the other tested tetramers. The proportional frequencies of KIR2DL2/3^+^ NK cells binding to the three tested HLA-C^*^03:04 tetramers were identical between YFV-vaccinated individuals or HIV-1- or HCV-infected individuals, and remained stable following YFV vaccination. These data demonstrate consistent hierarchies in the frequency of primary KIR2DL2/3^+^ NK cells binding HLA-C^*^03:04/peptide complexes that were determined by the HLA-C-presented peptide and not modulated by the underlying viral infection or vaccination.

## Introduction

Natural killer (NK) cells are an important component of the innate immune system and play a critical role in the early control of infections and malignancies. NK cells can recognize virus-infected or malignantly transformed cells through sensing the downregulation of human leukocyte class I (HLA-I) molecules on the cell surface, changes in the HLA class I-presented peptide repertoire, and upregulation of ligands for activating NK cell receptors (1). Function of NK cells is regulated through a balanced interplay between activating and inhibitory receptors. One of the major receptor families modulating NK cell function is the group of killer-cell immunoglobulin-like receptors (KIRs), which interact with HLA-I molecules expressed on nucleated cells (2). KIRs contain either two or three immunoglobulin-like domains in the extracellular region, and are named accordingly as KIR2D or KIR3D receptors. KIRs exhibiting a short cytoplasmic tail deliver an activating signal upon stimulation through interaction with the adaptor molecule DAP-12. Inhibitory KIRs possess a long cytoplasmic tail containing immunoreceptor tyrosine-based inhibitory motifs (ITIMs) and prevent NK cell activation after ligand binding (3). Several epidemiological studies have shown that expression of specific KIRs can be beneficial in the context of viral infections, especially when expressed in combination with their respective HLA-I ligands. As an example, the combination of KIR2DL3 and HLA-C alleles of the HLA-C group 1 (HLA-C1) was associated with spontaneous clearance of HCV infection (4, 5).

Multiple studies have shown that interactions between KIRs and HLA-I are not only determined by the HLA-I ligand but also modulated by the peptide presented by the respective HLA-I molecule (6–9). The resolution of crystal structures of inhibitory KIRs in complex with HLA-I/peptide complexes has further unraveled the importance of the sequence of HLA-I presented peptides, showing that KIR-binding is highly influenced by carboxyl-terminal residues of the HLA-I-presented peptides (10, 11). Several studies furthermore demonstrated that naturally occurring sequence mutations in virus-derived peptides are able to restore binding of HLA-I molecules to inhibitory KIRs, thereby preventing NK cell activation, and this has been suggested as a potential mechanism of viral escape from innate immune pressure (6–8, 12). NK cell receptor repertoires can also be shaped by viral infections, and expansion of NK cells expressing specific receptors has been observed in HCV, HCMV and HIV-1 infections, as well as in MCMV-infected mice (13–16). However, it remains unclear whether exposure to virus-derived HLA class I-presented peptides during infections or following vaccination has an impact on frequencies of KIR^+^ NK cells *in vivo*. To gain a better understanding of how viral infections and HLA-I-presented viral epitopes might influence frequencies of KIR^+^ NK cell subsets, we investigated the impact of YFV vaccination or HIV-1 and HCV infection on the frequencies of KIR2DL2/3^+^ NK cells binding to HLA-C^*^03:04/viral peptide complexes.

## Materials and methods

### Ethics statement

All study subjects provided written informed consent for participation under protocols approved by the Ärztekammer Hamburg.

### Study population

The demographics and clinical characteristics of study subjects are summarized in Table 1. Study subjects included: 5 individuals enrolled in a Yellow Fever Virus (YFV) vaccine study at the Bernhard Nocht Institute for Tropical Medicine. Peripheral blood samples were collected one day prior to vaccination and at day 1, day 3, day 5, and day 28 post vaccination. The samples were transported to the laboratory and processed directly afterwards. We furthermore obtained samples from 5 chronically HIV-1-infected individuals (on HAART) and 5 chronically HCV-infected individuals (treatment naïve). The samples were transported to the laboratory and processed immediately afterwards. Additionally PBMCs isolated from peripheral blood samples of healthy donors, recruited at the University Medical Center Hamburg-Eppendorf, were used.

**Table 1:** Study population.

**Cohort**	***n***	**Sex (female/male)**	**Age in years (mean with range)**	**Viral load in copies/ml (mean with range)**	**CD4 count (mean with range)**
YFV	5	4/1	31 (24–35)	n.a.	n.a.
HIV-1	5	1/4	58 (31–78)	242,000 (140,000–290,000)	353 (251–478)
HCV	5	1/4	49 (29–59)	2,950,000 (450,000–6,300,000)	n.a.

### MHC-I tetramer staining of primary human PBMCs

**Figure 1:**
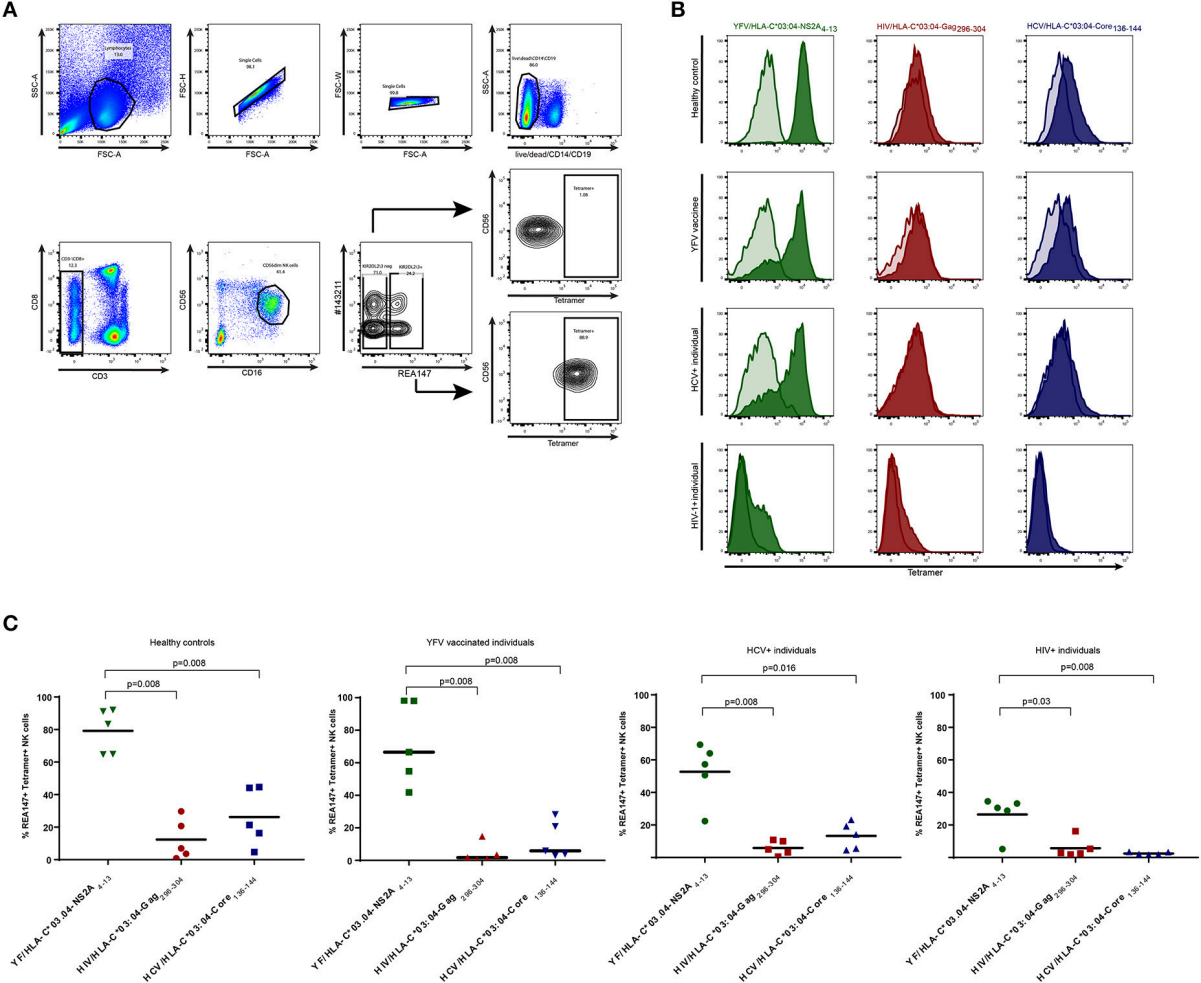
Frequencies of tetramer^+^ KIR2DL2/3^+^ NK cells in YFV vaccine-recipients, HIV-1-infected individuals, HCV-infected individuals and healthy controls. Staining of NK cells with YFV/HLA-C*03:04-NS2A_4−13_-, HIV/HLA-C*03:04-Gag_296−304_- and HCV/HLA-C*03:04-Core_136−144_-tetramers is depicted in green, red and blue respectively. **(A)** Gating strategy used to identify KIR2DL2/3^+^ (clone REA147^+^) and KIR2DL2/3^neg^ (clone REA147^neg^) NK cells. **(B)** Histograms demonstrating representative tetramer-staining of KIR2DL2/3^+^ NK cells (tinted) and KIR2DL2/3^neg^ NK cells (transparent) of healthy control subject, YFV vaccine recipient, HCV-infected individual and HIV-1-infected individual. **(C)** Scatter Plots of frequencies of tetramer^+^ KIR2DL2/3^+^ (clone REA147^+^) NK cells in all study groups. Black bar represents median of each group. Freshly isolated PBMCs were used for all study groups except HIV-1-infected individuals, for which cryopreserved PBMCs were used. *P*-values were calculated using Mann-Whitney test.

PE-labeled HLA-C^*^03:04 tetramers refolded with either the yellow fever virus-derived peptide HAVPFGLVSM (YFV/HLA-C^*^03:04NS2A_4−13_), the HIV-1-derived peptide YVDRFFKVL (HIV/HLA-C^*^03:04Gag_296−304_) and the HCV-derived peptide YIPLVGAPL (HCV/HLA-C03:04Core_136−144_) were provided by the NIH Tetramer Core Facility. Tetramers were used for staining of primary human PBMCs. Briefly, 1 × 10^6^ cells were stained for 30 min at 4°C with a mixture of live/dead marker, anti-CD14-BV510, anti-CD19-BV510, anti-CD8-PerCP-Cy5.5, anti-CD3-PB, anti-CD56-BUV395, anti-CD16-BV785, anti-KIR2DL3-APC (clone REA147, Miltenyi), anti-KIR2DL1-FITC (clone #143211, R&D Systems). All KIR antibodies used are shown in Supplementary Table 1. Cells were washed in sterile PBS containing 3% fetal bovine serum (FBS) and incubated twice on ice with 50 μl Blocking Buffer (sterile PBS + 10% human serum + 3% FBS) in a 96 well plate. After blocking, cells were stained with the corresponding tetramer at a 1/100 dilution in 50 μl Blocking Buffer resulting in a tetramer concentration of 11 ng/ml and incubated on ice for 60 min. After two washing steps cells were fixed with Fixation Buffer (4% paraformaldehyde in sterile PBS) and analyzed by flow cytometry (BD LSR Fortessa). Gates were set to only include live CD3^neg^ CD14^neg^ CD19^neg^ CD56^dim^ CD16^+^ KIR2DL2/3^+^ (defined by REA147 binding) NK cells, excluding all CD3^+^ CD14^+^ CD19^+^ KIR2DL2/3^neg^ cells (Figure 1A). Measurement of tetramer-binding was assessed as percentage of PE-positive cells.

### Assessment of binding affinities of peptides to HLA-C^*^03:04

For peptide titration experiments the corresponding peptides were synthesized (peptides & elephants GmbH, Hennigsdorf, Germany) and loaded on HLA-C^*^03:04-transfected 721.221 cells containing a CRISPR/Cas9 generated TAP knock out (221-TAPko-HLA-C^*^03:04). Cells were starved for 4 h in FBS-free RPMI 1640, and the corresponding peptides were added to the cell culture medium in concentrations of 0, 5, 10, 50, 100, 200, and 250 μM, and incubated for 21 h at 26°C. To determine HLA-C^*^03:04 stabilization, cells were stained with anti-pan-HLA-I-APC (Bioscience), washed, fixed, and analyzed by flow cytometry (BD LSR Fortessa). HLA-I stabilization was assessed as median MFI of APC and normalized to background expression.

### Assessment of binding affinity of KIR-Fc construct to HLA-C^*^03:04 peptide complex

For KIR-Fc staining 221-TAPko-HLA-C^*^03:04 cells pulsed with YFV/NSA2A_4−13_-, HIV/Gag_296−303_- or HCV/Core_136−144_-peptides were stained using KIR2DL3-Fc chimera (R&D) for 1 h on ice. After two washing steps with ice-cold 2% FBS/PBS cells were incubated with 1.25 μL of goat anti-hIgG(Fc)-PE antibody (Fisher Scientific). After washing with ice-cold 2% FBS/PBS cells were fixed with Fixation Buffer and analyzed using flow cytometry (BD LSR Fortessa). Measurement of KIR-Fc binding was assessed as percentage of PE-positive cells.

### Assessment of binding avidities of MHC-I tetramers to KIR2DL2/3

For tetramer titration experiments primary human PBMCs were stained with 1,100, 550, 110, 55, 11, 5, 1, and 0 ng/ml of the YFV/HLA-C^*^03:04-NS2A_4−13_-, the HIV/HLA-C^*^03:04-Gag_296−304_- and the HCV/HLA-C^*^03:04-Core_136−144_-tetramers for 1 h on ice after blocking with blocking buffer (sterile PBS+ 10% human serum+ 3% FBS). After two washing steps the cells were fixed with Fixation Buffer and analyzed using flow cytometry (BD LSR Fortessa). Measurement of tetramer binding was assessed as median MFI of PE expression and normalized to 100%.

### Functional assessment of tetramer-binding NK cell populations

For the functional assessment of tetramer^+^ and tetramer^neg^ KIR2DL3^+^ NK cell populations (defined as clone #180701 positive and clone #143211 negative), PBMCs were isolated from whole blood of one donor (donor 2) and rested overnight in R20 (RPMI 1640 containing 20% FBS) and 1 ng/ml IL-15. 1x10^6^ PBMCs per sample were stained with anti-KIR2DL3-APC (clone #180701) and anti-KIR2DL1-FITC (clone #143211) for 30 min at 4°C. The use of clone #180701 allowed for the specific gating on KIR2DL3+ NK cells as described previously (17). After washing, cells were incubated twice in Blocking Buffer (sterile PBS + 10% human serum + 3% FBS) and stained with 1/10 dilution of the YFV/HLA-C^*^03:04 NS2A_4−13_-tetramer. Tetramer-stained cells were subsequently co-incubated with 721.221 cells or 221-TAPko-HLA-C^*^03:04 cells pulsed with YFV/NSA2A_4−13_-, HIV/Gag_296−303_- or HCV/Core_136−144_-peptides. After 1 h incubation at 37°C, Golgistop (BD Bioscience) was added and cells were transferred to 26°C. Cells were incubated for 5 h, and surface staining was performed with a mixture of live/dead dye, anti-CD14-BV510, anti-CD19-BV510, anti-CD3-BV510, anti-CD8-PerCP-Cy5.5, anti-CD56-BUV395, anti-CD16-BV785 and anti-CD107a-BV421 (Biolegend). After two washing steps cells were fixed with Fixation Buffer (4% paraformaldehyde in sterile PBS) and analyzed using flow cytometry (BD LSR Fortessa). Gates were set to only include live CD3^neg^ CD14^neg^ CD19^neg^ CD56^dim^ CD16^+^ KIR2DL3^+^ NK cells, while CD3^+^ CD14^+^ CD19^+^ KIR2DL3^neg^ cells were excluded. Degranulation of NK cells was assessed as percentage of CD107a positive cells.

### Data analysis

Data analysis was performed using BD FACS Diva Software, FlowJo V10, Microsoft Excel 2017, GraphPad Prism 7 and Adobe Illustrator CC 2015.

### Data availability

Data used in this study have been collected in a clinical study and are subject to the regulation of the Ethics Committee of the Ärztekammer Hamburg that approved these studies. Participant's written consent has been provided to data generation and handling according to the approved protocols. Data storage is performed by the HPI. Data are available upon request and can be shared after confirming that data will be used within the scope of the originally provided informed consent.

## Results

### Consistent hierarchies of HLA-C^*^03:04 tetramer^+^ KIR2DL2/3^+^ NK cells in YFV vaccinees or HIV-1- and HCV-infected individuals

HLA-C tetramers have been previously reported to bind to primary human NK cells (18), but it remains unknown whether the frequencies of HLA-C tetramer-binding KIR2DL2/3^+^ NK cells change during viral infections or are influenced by antigen-exposure. The YFV-derived peptide NS2A_4−13_, the HIV-1-derived peptide Gag_296−304_ and the HCV-derived peptide Core_136−144_ were previously described to bind to HLA-C^*^03:04 and enable KIR2DL3-binding (7, 8, 17). To investigate whether frequencies of KIR2DL2/3^+^ NK cells binding to HLA-C^*^03:04 molecules presenting these peptides differ in YFV vaccine recipients or HIV-1- and HCV-infected individuals, we used HLA-C^*^03:04 tetramers refolded with the respective peptides as a tool to *ex vivo* stain primary human NK cells of YFV-vaccinated (28 days post vaccination), HIV-1-infected or HCV-infected individuals (Table 1). Stainings were performed using freshly isolated PBMCs for healthy controls, YFV vaccine recipients and HCV-infected individuals, or frozen PBMCs derived from HIV-1-infected individuals (Figure 1). While frequencies of tetramer^+^ KIR2DL2/3^+^ (clone REA147^+^) NK cells varied between the different study groups, the relative hierarchy of the respective tetramer^+^ NK cells did not differ between HIV-1-infected, HCV-infected, YFV-vaccinated or control individuals. YFV/HLA-C^*^03:04-NS2A_4−13_-tetramers consistently bound to the highest percentage of KIR2DL2/3^+^ NK cells, whereas HCV/HLA-C^*^03:04-Core_136−144_-tetramers and HIV/HLA-C^*^03:04-Gag_296−304_-tetramers bound to a significantly lower percentage of KIR2DL2/3^+^ NK cells (Figures 1B,C). Binding of KIR2DL3-Fc construct to 221-TAPko-HLA-C^*^03:04 pulsed with YFV/NSA2A_4−13_, HIV/Gag_296−304_, HCV/Core_136−144_ peptide, respectively, showed similar hierarchies (Supplementary Figure 1). The percentage of YFV/HLA-C^*^03:04-NS2A_4−13_-tetramer-binding KIR2DL2/3^+^ NK cells did furthermore not differ between individuals encoding for HLA-C^*^03 and HLA-C^*^03-negative individuals (in the 10 individuals from the YFV vaccine and healthy cohorts for which HLA class I typing was available, median of 74.2 vs. 78.8%, *p* > 0.9, Supplementary Figure 2). Taken together, these data show that KIR2DL2/3^+^ NK cells follow a consistent peptide-dependent hierarchy in their binding to HLA-C^*^03:04 tetramers, which is not influenced by whether a study subject encodes for HLA-C^*^03 and is furthermore independent of the underlying viral setting, suggesting a lack of antigen-dependent expansion of these NK cell populations. HLA-C group 1 tetramers, such as the HLA-C^*^03:04 tetramers used here, can therefore serve as a reagent to monitor the frequencies of KIR2DL2^+^ or KIR2DL3^+^ NK cells.

### Stable frequencies of YFV-specific tetramer^+^ KIR2DL2/3^+^ NK cells in YFV-vaccinated individuals over time

**Figure 2:**
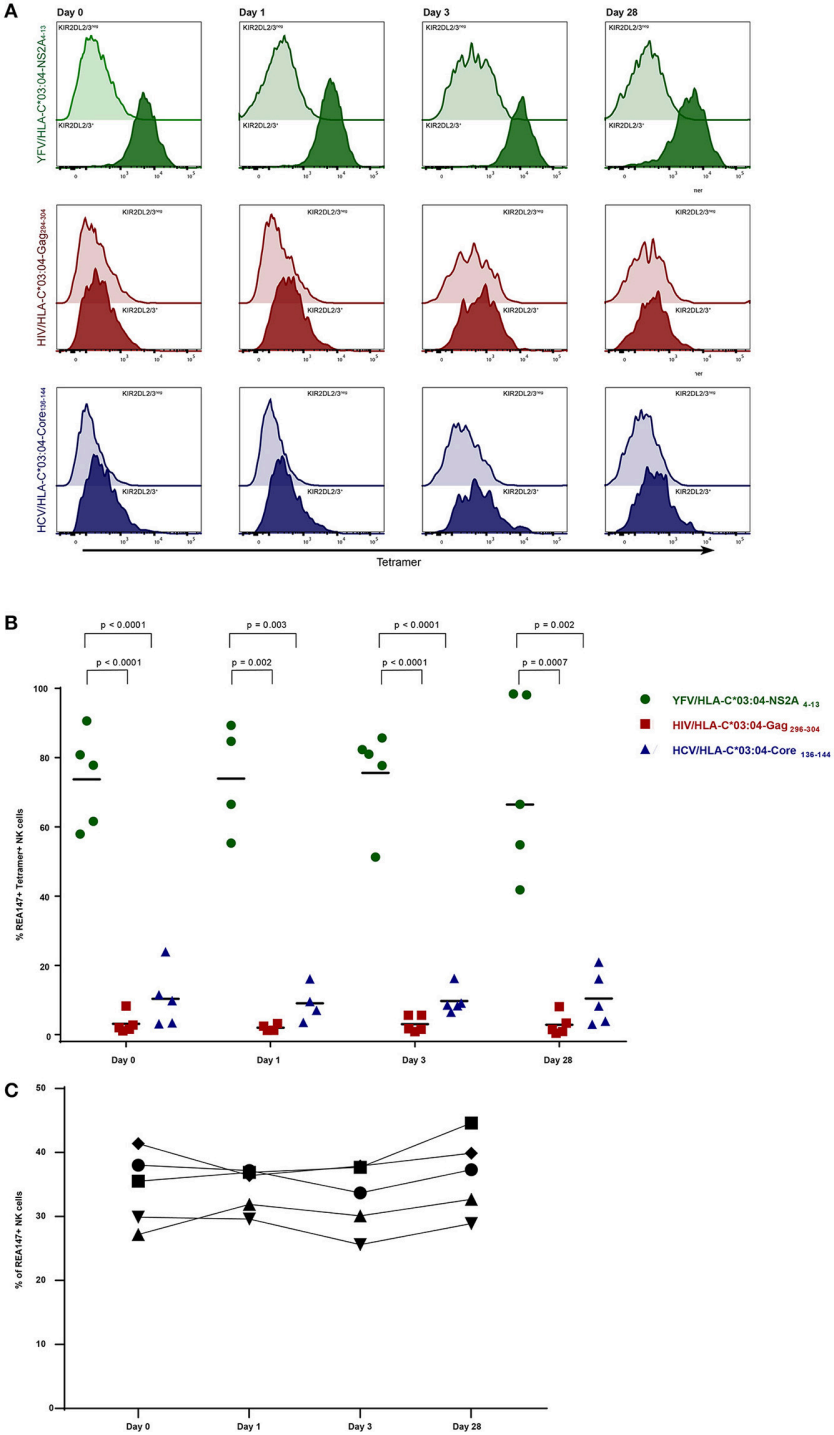
Frequencies of KIR2DL2/3^+^ tetramer^+^ NK cells in YFV vaccinees over time. Staining with YFV/HLA-C*03:04-NS2A_4−13_-, HIV/HLA-C*03:04-Gag_296−304_- and HCV/HLA-C*03:04-Core_136−144_- tetramers is depicted in green, red and blue, respectively. **(A)** Histograms demonstrating representative tetramer-staining of KIR2DL2/3^+^ (clone REA147^+^) NK cells (tinted) and KIR2DL2/3^neg^ NK cells (transparent) derived from one YFV vaccine recipient at 0, 1, 3, and day 28 after vaccination. **(B)** Scatter Plot of frequencies of tetramer^+^ KIR2DL2/3^+^ (clone REA147^+^) NK cells in the 5 YFV vaccine recipients stained with the respective tetramers at 0, 1, 3, and day 28. Black bar represents median of each group. **(C)** Frequencies of total KIR2DL2/3^+^ (clone REA147^+^) NK cells at 0, 1, 3, and day 28 after vaccination. Freshly isolated PBMCs were used for all experiments. *P*-values were calculated using Mann-Whitney test.

To assess whether KIR2DL2/3^+^ NK cells expanded following antigen challenge, we performed YFV/HLA-C^*^03:04-NS2A_4−13_-tetramer staining of primary PBMCs in 5 YFV-vaccinated individuals at 0, 1, 3, and day 28 of vaccination with YFV-17D. Stainings with HIV/HLA-C^*^03:04-Gag_296−304_- and HCV/HLA-C^*^03:04-Core_136−144_-tetramers were performed at the same times as controls. To control for a possible influence of HCMV infection on NK cell frequencies, vaccine recipients were tested for HCMV infection (3 individuals were positive and 2 negative for HCMV IgG or IgM, data not shown). No changes in the average frequency of YFV/HLA-C^*^03:04-NS2A_4−13_-tetramer^+^ KIR2DL2/3^+^ NK cells were observed following YFV-17D vaccination (Figure 2). Already before YFV vaccination, YFV/HLA-C^*^03:04-NS2A_4−13_-tetramers bound to the majority of KIR2DL2/3^+^ NK cells (mean 74%, range 57–90%), and did not significantly change following vaccination. The percentage of KIR2DL2/3^+^ NK cells binding either the HIV/HLA-C^*^03:04-Gag_296−304_- or the HCV/HLA-C^*^03:04-Core_136−144_-tetramer also did not change following vaccination (Figures 2A,B). In addition, the overall frequency of KIR2DL2/3^+^ NK cells did not change following YFV vaccination (Figure 2C). Again, frequencies of tetramer-positive KIR2DL2/3^+^ NK cells did not differ between individuals encoding for HLA-C^*^03 and not encoding for HLA-C^*^03 (data not shown). These data demonstrate that the percentages of KIR2DL2/3^+^ NK cells binding HLA-C^*^03:04/peptide complexes are determined by the HLA-I-presented peptide, and that these percentages do not change following YFV vaccination.

### Peptide-dependent hierarchies of tetramer^+^ KIR2DL2/3^+^ NK cells are associated with the binding affinity of HLA-C^*^03:04/peptide complexes to KIR2DL2/3

**Figure 3:**
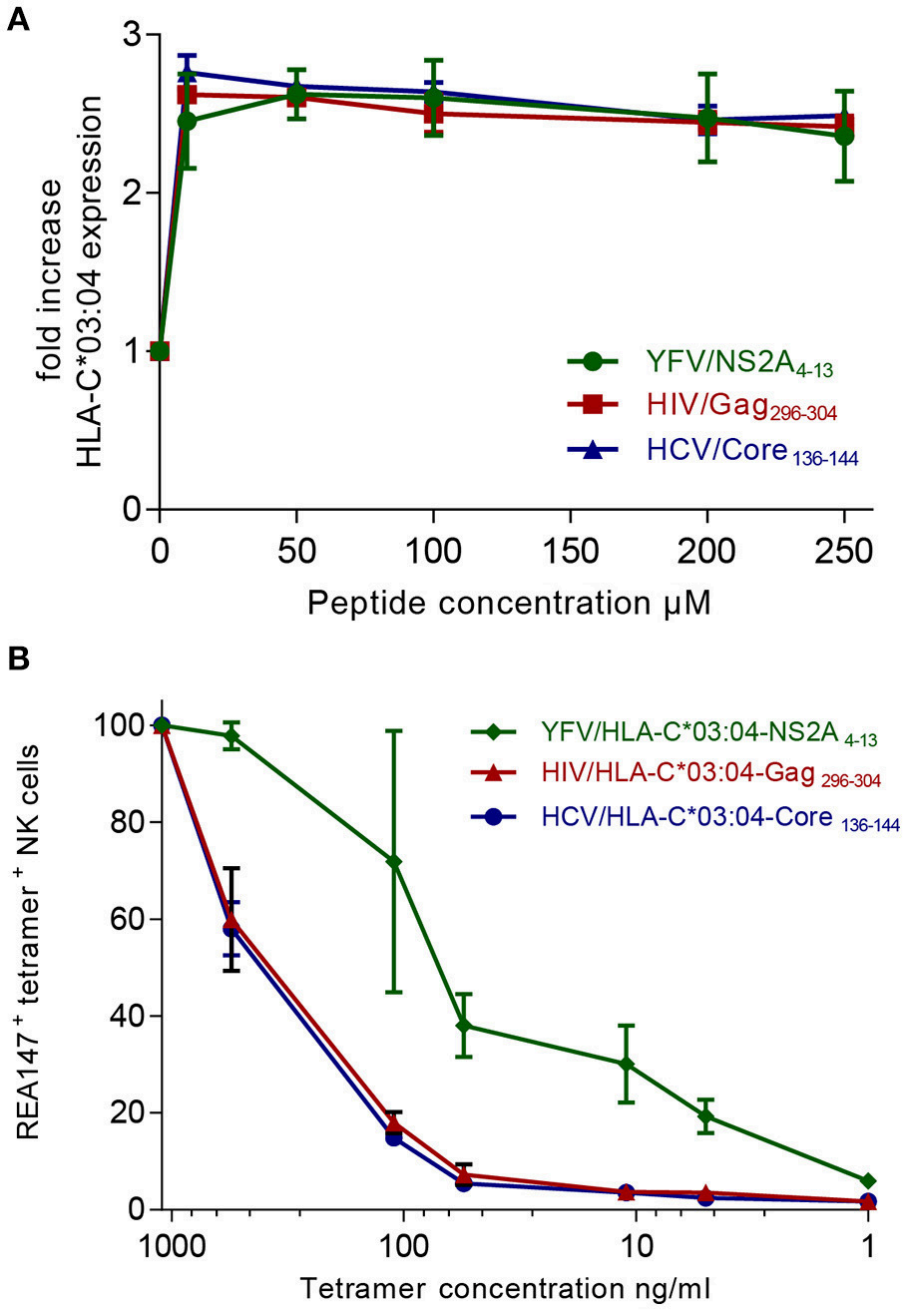
The YFV/HLA-C*03:04-NS2A_4−13_-tetramer exhibits the strongest binding affinity to KIR2DL2/3. **(A)** Fold increase of HLA-C*03:04 expression on 221-TAPko-HLA-C*03:04 cells incubated with the indicated concentrations of the YFV/NSA2A_4−13_ (green), the HIV/Gag_296−303_ (red), and HCV/Core_136−144_ (blue) peptides is shown. **(B)** Percentage of tetramer^+^ KIR2DL2/3^+^ (clone REA147^+^) NK cells stained with YFV/HLA-C*03:04-NS2A_4−13_-, HIV/HLA-C*03:04-Gag_296−304_- and HCV/HLA-C*03:04-Core_136−144_-tetramers are shown at the indicated tetramer concentrations.

To dissect whether the observed hierarchies of HLA-C^*^03:04/peptide^+^ KIR2DL2/3^+^ NK cells were influenced by the affinity of either the respective peptide to the HLA-C^*^03:04 molecule or the HLA-C^*^03:04/peptide complex to KIR2DL2/3, we performed *in vitro* assays investigating the binding affinity of the peptides YFV/NSA2A_4−13_, HIV/Gag_296−303_, HCV/Core_136−144_ to HLA-C^*^03:04, and the binding avidity of the respective YFV/HLA-C^*^03:04-NS2A_4−13_-, HIV/HLA-C^*^03:04-Gag_296−304_- and HCV/HLA-C^*^03:04-Core_136−144_-tetramers to KIR2DL2/3. For assessment of binding affinities of YFV/NSA2A_4−13_-, HIV/Gag_296−303_-, HCV/Core_136−144_-peptides to HLA-C^*^03:04, we pulsed .221 cells, deficient of classical HLA class I molecules and TAP, but transfected with HLA-C^*^03:04 (221-TAPko-HLA-C^*^03:04), with increasing concentrations of the respective peptides. All three peptides showed similar binding affinities to HLA-C^*^03:04, which peaked at 10 μM and exhibited saturation for concentrations >50 μM (Figure 3A). The binding data were in line with predicted HLA-C^*^03:04 binding scores using the NetMHCpan 4.0 program, which showed similar scores for all three peptides ranging from 0.3 to 0.5 (19). These data suggested that peptide-binding affinities to HLA-C^*^03:04 did not explain the observed hierarchies of HLA-C^*^03:04/peptide^+^ KIR2DL2/3^+^ NK cells. We next assessed binding of HLA-C^*^03:04/peptide-tetramers to primary KIR2DL2/3^+^ NK cells. The YFV/HLA-C^*^03:04-NS2A_4−13_-tetramer showed a significantly stronger avidity to NK cells expressing KIR2DL2/3 compared to HIV/HLA-C^*^03:04-Gag_296−304_- and HCV/HLA-C^*^03:04-Core_136−144_-tetramers (p = 0.005), which both bound to a similar extent (Figure 3B). Taken together, these data are consistent with a model in which the observed hierarchical binding of YFV/HLA-C^*^03:04-NS2A_4−13_-, HIV/HLA-C^*^03:04-Gag_296−304_- and HCV/HLA-C^*^03:04-Core_136−144_-tetramers to KIR2DL2/3^+^ NK cells is determined by the binding affinity of the HLA-C^*^03:04/peptide complexes to KIR2DL2/3.

### Higher functional capacity of tetramer^+^ KIR2DL3^+^ NK cell subpopulations is inhibited through binding to HLA-I/peptide complex

**Figure 4:**
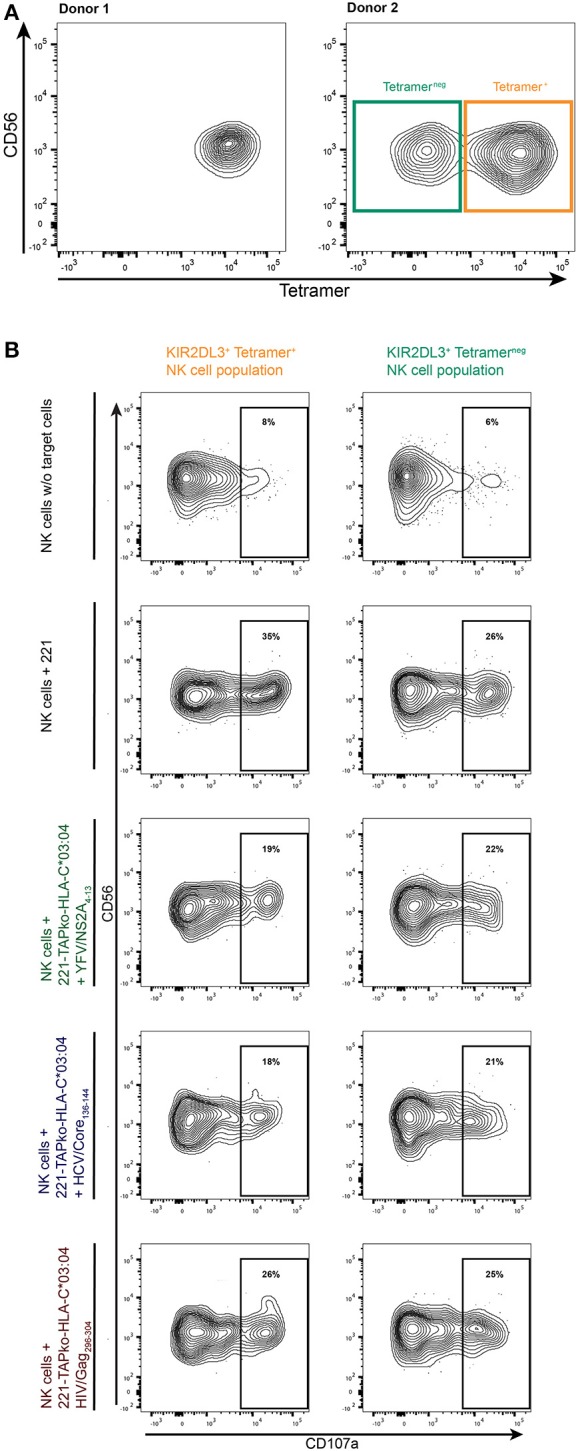
Functional determination of tetramer^+^ and tetramer^neg^ KIR2DL3^+^ NK cell subpopulations. **(A)** YFV/HLA-C*03:04-NS2A_4−13_-tetramer binding to KIR2DL3^+^ (clone #180701) NK cells in two donors. The right flow plot shows two KIR2DL3^+^ NK cell populations either binding the YFV/HLA-C*03:04-NS2A_4−13_-tetramer (orange, tetramer^+^) or not (teal, tetramer^neg^) in donor 2. **(B)** PBMCs from donor 2 were incubated with either no other cells, 221 cells, or 221-TAPko-HLA-C*03:04 cells pulsed with YFV/NSA2A_4−13_- (green), HCV/Core_136−144_- (blue) and HIV/Gag_296−303_- (red) peptide. The gates show the percentage of CD107a+ from either YFV/HLA-C*03:04-NS2A_4−13_-tetramer^+^ (orange) or tetramer^neg^ (teal) KIR2DL3^+^ (clone #180701) NK cells.

While most study subjects exhibited one principal population of YFV/HLA-C^*^03:04-NS2A_4−13_-tetramer^+^ NK cells (Figure 4A, donor 1), some study subjects exhibited both YFV/HLA-C^*^03:04-NS2A_4−13_-tetramer^+^ and YFV/HLA-C^*^03:04-NS2A_4−13_-tetramer^neg^ KIR2DL3^+^ NK cell populations (Figure 4A, donor 2). This provides an opportunity to compare the functional activity of tetramer^+^ and tetramer^neg^ KIR2DL3^+^ NK cells, using an antibody previously described as being KIR2DL3-specific (clone #180701) (17). To investigate whether binding of the YFV/HLA-C^*^03:04-NS2A_4−13_-tetramer was associated with enhanced functionality of KIR2DL3^+^ NK cells, we compared the ability of YFV/HLA-C^*^03:04-NS2A_4−13_-tetramer^+^ and -tetramer^neg^ KIR2DL3^+^ NK cell subpopulations in donor 2 to degranulate in response to classical HLA-I deficient 721.221 cells (.221) (Figure 4B). PBMCs were stained with the YFV/HLA-C^*^03:04-NS2A_4−13_-tetramer and co-incubated with .221 cells or .221-TAPko-HLA-C^*^03:04 cells pulsed with either YFV/NSA2A_4−13_-, HIV/Gag_296−303_- or HCV/Core_136−144_-peptide. YFV/HLA-C^*^03:04-NS2A_4−13_-tetramer^+^ KIR2DL3^+^ NK cells expressed higher levels of CD107a following stimulation with HLA-I deficient .221 cells compared to the YFV/HLA-C^*^03:04-NS2A_4−13_-tetramer^neg^ KIR2DL3^+^ NK cell population (35 vs. 26%). The higher levels of CD107a-expression of YFV/HLA-C^*^03:04-NS2A_4−13_-tetramer^+^ KIR2DL3^+^ NK cells was however reverted when KIR2DL3^+^ NK cells were co-incubated with peptide-pulsed 221-TAPko-HLA-C^*^03:04 cells. YFV/NSA2A_4−13_- and HCV/Core_136−144_-peptide pulsed 221-TAPko-HLA-C^*^03:04 cells inhibited YFV/HLA-C^*^03:04-NS2A_4−13_-tetramer^+^ KIR2DL3^+^ NK cell degranulation strongest (19 vs. 35% (YFV) and 18 vs. 35% (HCV), respectively, whereas HIV/Gag_296−303_-peptide pulsed 221-TAPko-HLA-C^*^03:04 cells had the lowest ability to reduce degranulation (26 vs. 35%) (Figure 4B). In contrast, no effect of peptide-pulsed 221-TAPko-HLA-C^*^03:04 cells was observed on the degranulation of YFV/HLA-C^*^03:04-NS2A_4−13_-tetramer^neg^ KIR2DL3^+^ NK cell populations, which remained constant at around 25% (range 21–25%, Figure 4B, right column). These data show that the ability of KIR2DL3^+^ NK cells to bind YFV/HLA-C^*^03:04-NS2A_4−13_-tetramers was associated with a higher functional capacity of these cells, consistent with NK cell licensing, and that engagement of the respective HLA-C^*^03:04/peptide ligand can inhibit KIR2DL3^+^ NK cell function.

## Discussion

Inhibitory KIRs expressed on NK cells bind to HLA class I molecules, and this interaction is modulated by the HLA class I-presented peptide. However, it is not known whether peptide-dependent KIR/HLA interactions provide a peptide-specific signal to NK cells and modulate frequencies of KIR^+^ NK cells. We investigated the impact of viral infections and vaccination on frequencies of HLA-C^*^03:04/peptide-binding KIR2DL2/3^+^ NK cell populations and their function. Cross-sectional studies of HCV- and HIV-1-infected individuals and longitudinal studies in YFV vaccine recipients showed that frequencies of HLA-C^*^03:04/peptide-binding KIR2DL2/3^+^ NK cells in peripheral blood were not influenced by whether a study subject encoded for HLA-C^*^03, and were furthermore independent of the underlying viral infection and did not change following YFV vaccination. Rather, hierarchies of YFV/HLA-C^*^03:04-NS2A_4−13_-, HIV/HLA-C^*^03:04-Gag_296−304_- and HCV/HLA-C^*^03:04-Core_136−144_-tetramers^+^ KIR2DL2/3^+^ NK cells in these different infections and following vaccination were determined by the affinity of KIR2DL2/3 to the respective HLA-C^*^03:04/peptide complex. HLA-C group 1 tetramers, such as the HLA-C^*^03:04 tetramers used here, can therefore serve as a reagent to monitor the frequencies of KIR2DL2^+^ or KIR2DL3^+^ NK cells over time.

Several studies demonstrated that interactions between HLA class I molecules and their KIR ligands are influenced by the sequence of the peptide that is presented by the respective HLA-I molecule. Crystal structures of KIR2DL2 and KIR3DL1 in complex with HLA-I/peptide complex provided structural evidence that KIR/HLA-I interactions are highly susceptible to changes in the C-terminal sequence of HLA-I-presented peptides (10, 11). While the peptide-dependence of these interactions is well established, it remains unknown whether HLA-I-presented peptides can induce expansion or contraction of KIR^+^ NK cell populations binding HLA-I/peptide complexes. Using HLA-C^*^03:04/peptide tetramer-staining of primary human KIR2DL2/3^+^ NK cells derived from healthy participants in a YFV vaccine trial and from HCV- and HIV-1-infected individuals, we tested the hypothesis that KIR2DL2/3^+^ NK cells binding to HLA-C^*^03:04 molecules presenting a specific viral peptide would differ in frequency between the different study groups. The study cohorts included individuals carrying or lacking HLA-C^*^03, and the selected peptides were previously shown to be naturally processed and presented by HLA class I, as indicated by described specific CD8+ T cell responses against the tested epitopes (7, 20, 21). The results from this study did not support the initial hypothesis, but rather show that KIR2DL2/3^+^ NK cells follow a consistent peptide-dependent hierarchy in their binding to HLA-C^*^03:04, which is independent of the underlying viral infection or vaccination, and also of whether a study subject encoded for HLA-C^*^03 or not, but was determined by the binding affinity of the respective HLA-C^*^03:04/peptide complex to KIR2DL2/3. These findings are in line with a previous study comparing binding of HLA-B57 tetramers refolded with a Dengue virus (DENV)-derived peptide in DENV-infected vs. uninfected individuals, demonstrating comparable frequencies of HLA-B57/DENV peptide tetramer^+^ CD56^dim^ NK cells (22). Furthermore, Colantonio et al. observed staining of KIR2D^+^ NK cells with tetramers refolded by the SIV-derived peptide GAG_71−79_ GY9 in a previously non-SIV-exposed rhesus macaque (23). These data show that HLA-C group 1/peptide tetramers can be used to identify KIR2DL2/3+ NK cell populations, but also suggest that an antigen-dependent expansion or contraction of the respective NK cell populations does not occur.

Expansion of individual NK cell subsets was reported for different viral infections. This included expansion of NK cells expressing Ly49H in MCMV-infected mice (16), NKG2C in HCMV- and HCV-infected individuals (13, 24), and KIR3DS1 in acute HIV-1 infections (15). Furthermore, contraction of NK cells was shown to occur in infections with HIV-1, HCV, and varicella zoster virus (25–27). However, the molecular mechanisms driving these expansions or contractions remain elusive, with some studies suggesting a role of licensing through interactions between HLA-I and inhibitory KIRs (15, 28). To examine whether KIR2DL2/3 are able to mediate NK cell expansion or contraction *in vivo*, we longitudinally assessed the frequency of KIR2DL2/3^+^ NK cells able to bind a YFV-derived peptide presented by HLA-C^*^03:04 in individuals receiving the YFV-17D vaccine. In contrast to a study reporting an increased expression of KIR2DL3 mRNA following vaccination with YFV-17D at day 7 (29), we observed stable frequencies of both total KIR2DL2/3^+^ NK cells as well as tetramer^+^ KIR2DL2/3^+^ NK cells for all four tested time points. The described gene induction of *KIR2DL3* after YFV vaccination might therefore have been restricted to other KIR-expressing cell populations, such as T cells, as only bulk KIR2DL3 mRNA levels on whole blood were quantified in the study by Gaucher et al. As previous studies also suggested an influence of HCMV infection on NK cell expansion (14, 16, 30), we assessed the HCMV status of our study subjects, and did not observe any differences in frequencies of tetramer^+^ KIR2DL2/3^+^ NK cells between HCMV^+^ and HCMV^neg^ individuals. As expansion of NK cells following viral infections has been largely described for activating NK cell receptors, including KIR3DS1 and NKG2C in humans and Ly49H in mice (14, 15, 30, 31), the capacity of NK cells to expand might be attributed to DAP-12, the adaptor molecule mediating signal transduction of activating receptors (30). Signaling of inhibitory receptors in contrast is mediated through ITIMs located in the intracellular tail of the receptor (32). Notably these expansions are not purely dependent on KIR-HLA interactions, as in TAP-deficient patients an expansion of NKG2C+ NK cells was observed albeit the very low levels of HLA class I (33). Our data suggest that engagement of the inhibitory receptor KIR2DL2/3 by HLA-C^*^03:04/peptide complexes is not inducing KIR2DL2/3^+^ NK cell expansion. This is further supported by the observation that frequencies of HLA-C^*^03:04/peptide complex-binding KIR2DL2/3^+^ NK cells did not differ between HLA-C^*^03^+^ and HLA-C^*^03^*neg*^ individuals. However, these data need to be interpreted in the context of the modest size of the study cohorts used, and confirmatory studies in larger cohorts are required to better control for additional factors that may influence KIR^+^ NK cell frequencies, such as specific KIR2DL2/3 and HLA-C subtypes, as well as CMV serostatus. Furthermore, not all KIR2DL2/3^+^ NK cells bound to HLA-C^*^03:04/peptide tetramers, suggesting that additional factors, such as KIR2DS2 genotype, surface KIR-expression levels or the ability of KIRs to cluster on the cell surface might impact HLA-C binding.

The results from our study show that binding of KIR^+^ NK cells to HLA-C^*^03:04/peptide complex is impacted by the biochemical properties of the HLA-I-bound peptide. We determined the binding affinities of the different tested peptides to HLA-C^*^03:04 and the avidity of HLA-C^*^03:04/peptide tetramers to KIR2DL2/3, and observed that the higher binding of YFV/HLA-C^*^03:04-NS2A_4−13_- compared to HIV/HLA-C^*^03:04-Gag_296−304_- and HCV/HLA-C^*^03:04-Core_136−144_-tetramers to KIR2DL2/3^+^ NK cells was associated with the higher binding avidity of YFV/HLA-C^*^03:04-NS2A_4−13_ complexes to KIR2DL2/3. This observation is in line with a previous study mapping binding-avidity and peptide-selectivity of Mamu-KIR3DL05 to the same domain of the receptor, thereby emphasizing the influence of the HLA-I bound peptide on KIR avidity (23). Furthermore, our results show that the binding of YFV/HLA-C^*^03:04-NS2A_4−13_-, HIV/HLA-C^*^03:04-Gag_296−304_- and HCV/HLA-C^*^03:04-Core_136−144_-tetramers to KIR2DL2/3^+^ NK cells was independent of the binding affinity of the corresponding peptide to HLA-C^*^03:04. Binding of inhibitory KIRs to HLA class I molecules during NK cell development has been shown to play a critical role in determining the functionality of KIR^+^ NK cells, a process referred to as NK cell licensing or education (34, 35). To assess the relevance of KIR2DL3^+^/HLA-C^*^03:04 interactions for the functionality of KIR2DL3^+^ NK cells, we compared the ability of tetramer^+^ and tetramer^neg^ KIR2DL3^+^ NK cells to degranulate in one individual, using the described KIR2DL3-specific antibody (clone #180701) (17). The results showed that the ability of KIR2DL3^+^ NK cells to bind to YFV/HLA-C^*^03:04-NS2A_4−13_-tetramers correlated with a higher functional capacity of these cells, which is consistent with NK cell licensing/education. We furthermore observed that the capacity of tetramer^+^ KIR2DL3^+^ NK cells was influenced by the engagement of KIR2DL3 to its ligand. Around 35% of tetramer^+^ KIR2DL3^+^ NK cells degranulated in response to classical HLA-I deficient 221 cells. When these tetramer^+^ KIR2DL3^+^ NK cells were exposed to 221 cells presenting either the YFV/NS2A_4−13_- or the HCV/Core_136−144_-peptide via HLA-C^*^03:04, degranulation was reduced, while 221 cells presenting the HIV/Gag_296−303_-peptide inhibited NK cell degranulation less. These data were in line with the low binding of HIV/HLA-C^*^03:04-Gag_296−304_-tetramers to KIR2DL3^+^ NK cells. We furthermore observed a tetramer^+^ and tetramer^neg^ population within KIR2DL3^+^ NK cells, which might suggest differential HLA-C^*^03:04-binding to different KIR2DL3 subtypes, as most NK cells will only express one of the two alleles (36). However, one limitation of studying KIR^+^ NK cells is the cross-reactivity of several of the available antibodies, in particular between closely related activating and inhibitory KIRs. While the antibody used to identify KIR2DL3^+^ NK cells (clone #180701) was described not to cross-react with KIR2DL2 and KIR2DS2 (17), we cannot completely rule out cross-reactivity with these receptors. In conclusion, the findings in this study demonstrate consistent hierarchies in the frequencies of KIR2DL2/3^+^ NK cells binding HLA-C^*^03:04/peptide complexes that were not changed by an underlying viral infection or vaccination.

## Author contributions

MZ and SL performed experiments and analyzed the data. FG, JS, CH, and AR recruited patients and processed patient materia. WG-B helped with assay design. SL and MA designed the study. MZ, SL, and MA wrote the manuscript. All authors provided continuous critical review of the data and commented on the manuscript.

### Conflict of interest statement

The authors declare that the research was conducted in the absence of any commercial or financial relationships that could be construed as a potential conflict of interest.

## Supplementary material

Supplementary Figure 1 Binding of KIR2DL3-Fc to GKL, GAL, YF, HIV-1 and HCV peptide pulsed 221-TAPko-HLA-C^*^03:04 cells.

Supplementary Figure 2 Frequency of KIR2DL2/3+ YFV/HLA-C^*^03:04NS2A_4−13_ NK cells in HLA-Cw3 and not-HLA-Cw3 donors.

Supplementary Table 1 Anti-KIR antibodies used in this study.
