# Supplementary figures and images for: Stable Frequencies of HLA-C*03:04/Peptide-Binding KIR2DL2/3+ Natural Killer Cells Following Vaccination
Source: Front Immunol. 2018 Oct 17;9:2361. doi: 10.3389/fimmu.2018.02361 (PMC6199360; doi:10.3389/fimmu.2018.02361)

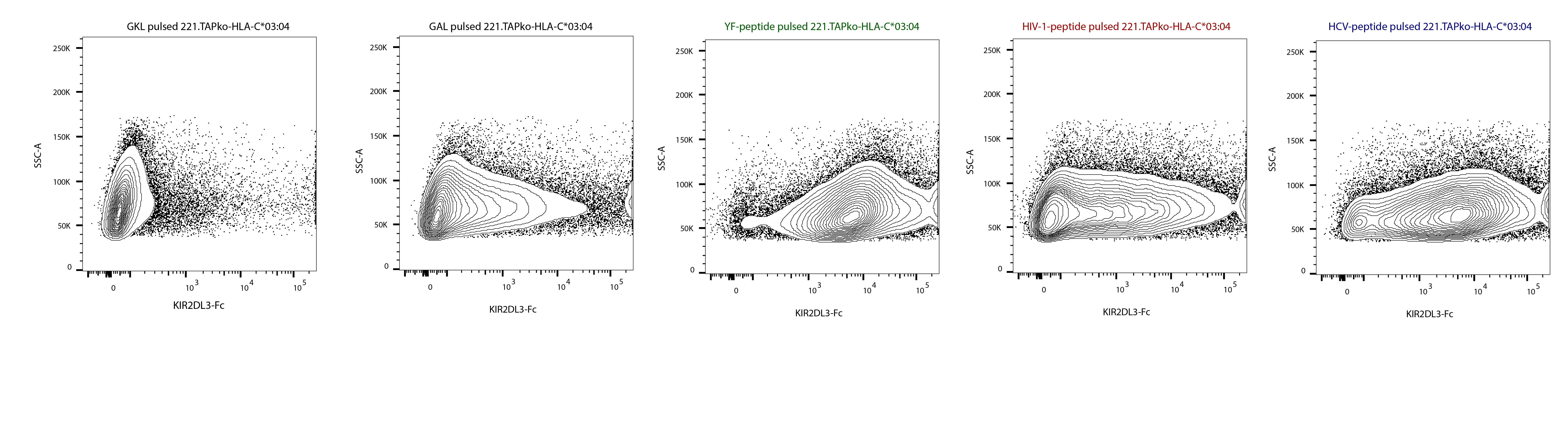

Supplement: Supplementary Figure 1 — Binding of KIR2DL3-Fc to GKL, GAL, YF, HIV-1 and HCV peptide pulsed 221-TAPko-HLA-C*03:04 cells. [file Image_1.TIF]

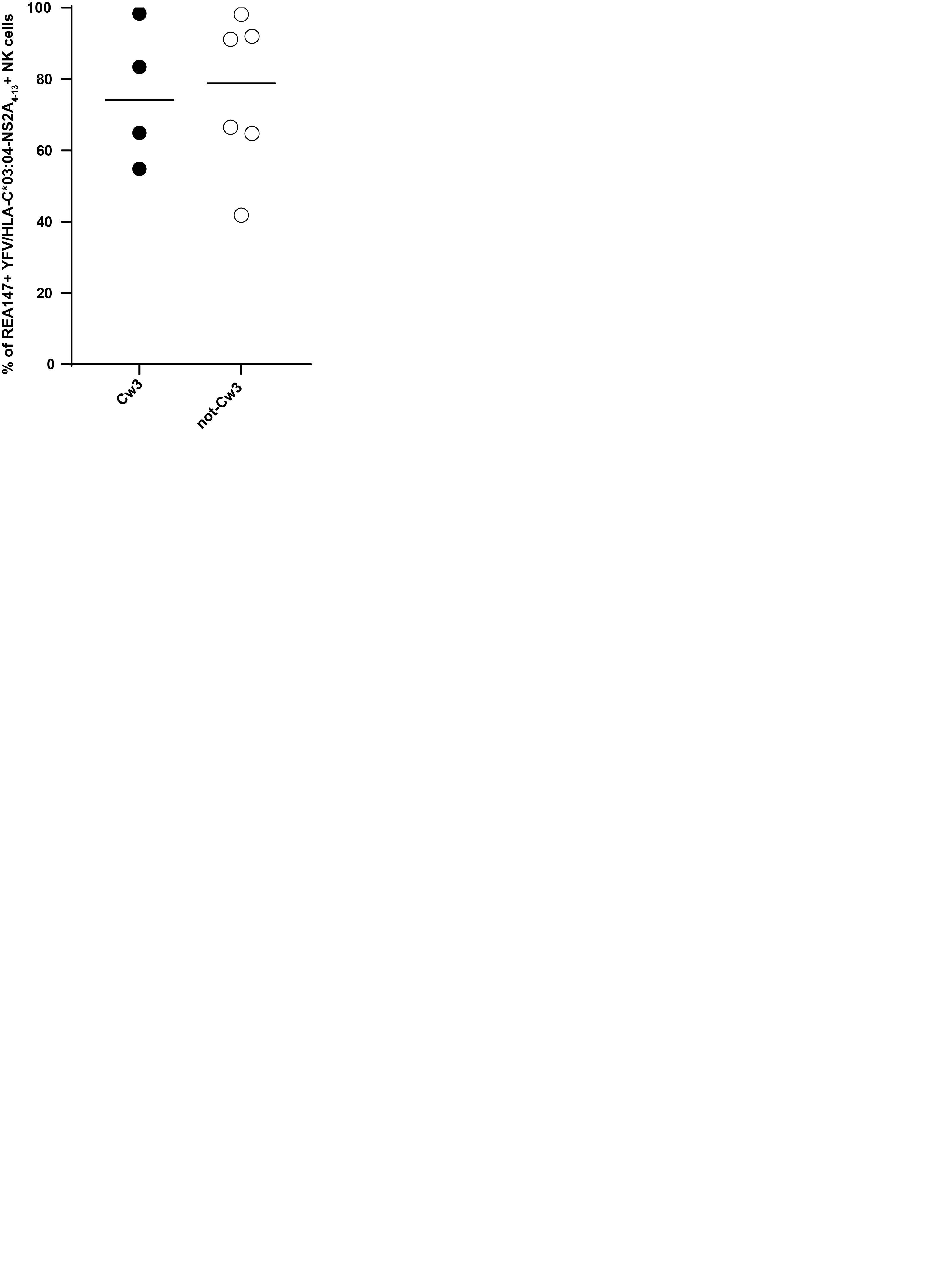

Supplement: Supplementary Figure 2 — Frequency of KIR2DL2/3+ YFV/HLA-C*03:04NS2A4−13 NK cells in HLA-Cw3 and not-HLA-Cw3 donors. [file Image_2.TIF]
