# Supplementary material for: Stable Frequencies of HLA-C*03:04/Peptide-Binding KIR2DL2/3+ Natural Killer Cells Following Vaccination
Source: Front Immunol. 2018 Oct 17;9:2361. doi: 10.3389/fimmu.2018.02361 (PMC6199360; doi:10.3389/fimmu.2018.02361)
Supplement: Supplementary Table 1 — Anti-KIR antibodies used in this study. [file Table_1.pdf]

Antibody	clone	company	reported cross reactivity
Human KIR/CD158 PE-conjugated antibody	#180704	R&D	2DL2, 2DS2, 2DS4
Human KIR2DL1/KIR2DS5 Fluorescein-conjugated antibody	#143211	R&D	2DS5
Human KIR2DL3/CD158b2 APC-conjugated antibody	#180701	R&D	
Human KIR2DL3/CD158b2 PE-conjugated antibody	#180701	R&D	
Human CD158b2 (KIR2DL3) PE-conjugated antibody	REA147	Miltenyi	2DL2
